# Supplementary figures and images for: Citrus junos Tanaka Peel Extract and Its Bioactive Naringin Reduce Fine Dust-Induced Respiratory Injury Markers in BALB/c Male Mice
Source: Nutrients. 2022 Mar 5;14(5):1101. doi: 10.3390/nu14051101 (PMC8912745; doi:10.3390/nu14051101)

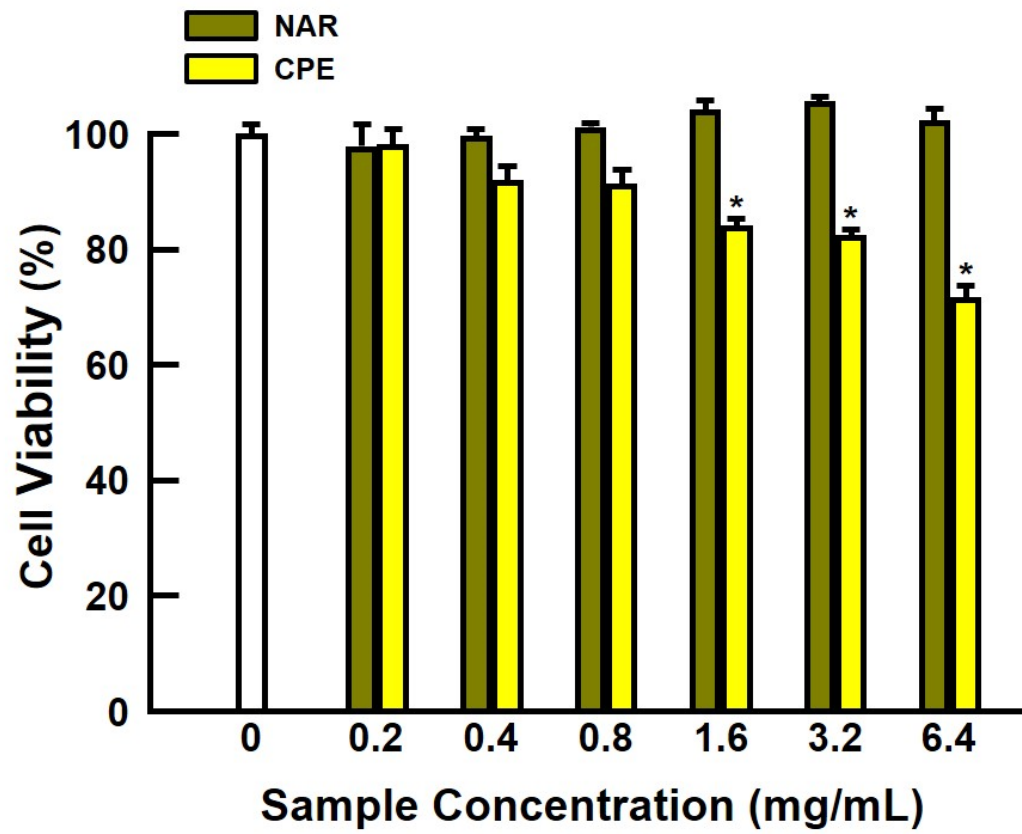

Figure S1. Cytotoxicity of CPE and NAR treatment on NCI-H460 cell line.

Supplement: Supplementary file 1 [file nutrients-14-01101-s001.zip › nutrients-1617686-supplementary.pdf]
